# Dimerization of the transmembrane domain of amyloid precursor proteins and familial Alzheimer's disease mutants

**DOI:** 10.1186/1471-2202-9-17

**Published:** 2008-01-30

**Authors:** Paul M Gorman, Sanguk Kim, Meng Guo, Roman A Melnyk, Joanne McLaurin, Paul E Fraser, James U Bowie, Avijit Chakrabartty

**Affiliations:** 1 Ontario Cancer Institute and Department of Medical Biophysics, University of Toronto, Toronto, Ontario, M5G 2M9, Canada; 2 Department of Life Science, Pohang University of Science and Technology, 790-784, South Korea; 3 Department of Microbiology and Molecular Genetics, Harvard, Boston, Massachusetts 02115, USA; 4 Center for Research in Neurodegenerative Disease, Department of Laboratory Medicine and Pathobiology, University of Toronto, Toronto, Ontario, M5S 3H2, Canada; 5 Department of Chemistry and Biochemistry, UCLA, Los Angeles, CA, 90095, USA

## Abstract

**Background:**

Amyloid precursor protein (APP) is enzymatically cleaved by γ-secretase to form two peptide products, either Aβ40 or the more neurotoxic Aβ42. The Aβ42/40 ratio is increased in many cases of familial Alzheimer's disease (FAD). The transmembrane domain (TM) of APP contains the known dimerization motif GXXXA. We have investigated the dimerization of both wild type and FAD mutant APP transmembrane domains.

**Results:**

Using synthetic peptides derived from the APP-TM domain, we show that this segment is capable of forming stable transmembrane dimers. A model of a dimeric APP-TM domain reveals a putative dimerization interface, and interestingly, majority of FAD mutations in APP are localized to this interface region. We find that FAD-APP mutations destabilize the APP-TM dimer and increase the population of APP peptide monomers.

**Conclusion:**

The dissociation constants are correlated to both the Aβ42/Aβ40 ratio and the mean age of disease onset in AD patients. We also show that these TM-peptides reduce Aβ production and Aβ42/Aβ40 ratios when added to HEK293 cells overexpressing the Swedish FAD mutation and γ-secretase components, potentially revealing a new class of γ-secretase inhibitors.

## Background

Currently, almost 4.5 million individuals in the United States have Alzheimer's disease (AD) and this number is expected to increase to approximately 16 million by 2050 [1]. Among these millions, 5–10% are a heritable form of the disease called familial AD (FAD). A subset of FAD is caused by mutations in the gene encoding for amyloid precursor protein (APP). APP degradation results in the formation of senile plaques, which are dense extracellular deposits localized to the limbic and association cortices and composed mainly of two amyloid peptides (Aβ40 and Aβ42) that are produced through proteolytic processing by β and γ secretases [2]. First β-secretase cleaves the APP extracellular juxtamembrane domain, and then γ-secretase cleaves the APP transmembrane domain (TM) either after Val711 or after Ala713 producing Aβ40 and Aβ42, respectively. In normal individuals APP processing leads to low Aβ42/40 ratios, while individuals with FAD mutations in the APP-TM domain have an increased Aβ42/40 ratio and therefore an increased proportion of the more neurotoxic Aβ42 [3].

Several lines of evidence indicate that APP dimerizes in its native membrane environment. Chemical crosslinking has indicated that APP can dimerize and use of an obligate APP dimer (with disulfide-linkage at the TM domains) shows that dimeric APP is efficiently cleaved by γ-secretase [4]. The APP-TM domain contains the motif GXXXG/A that is known to mediate dimerization of transmembrane helices in oligomeric membrane proteins [5]. Given that more than half of the mutations in APP that cause FAD are localized to the TM domain [2], we hypothesized that these mutations are capable of perturbing the dimerization and this leads to the increased Aβ42/40 ratio seen in FAD.

To test this hypothesis, we have examined synthetic peptides corresponding to TM segments of APP (APP-TM peptides) in detergent micelles and phospholipid bilayers. We have found that this domain is capable of dimerization, and FAD mutations within the APP-TM domain affect the dimerization propensity. We find that the dimerization equilibrium constants of the APP-TM variants measured *in vitro *are correlated to two well known clinical pathological features of AD patients. The constants are correlated to the average age of onset of AD symptoms and to the Aβ42/Aβ40 ratios observed in FAD. We demonstrate that the addition of our synthetic APP-TM peptides to cell culture models reduces the production of both Aβ40 and Aβ42. We propose that APP-TM mutations increase monomerization, which leads to the increased Aβ42/40 ratios seen in FAD cell culture models.

## Results

### Characterization of secondary structure and oligomerization state of APP-TM peptides

**Figure 1:**
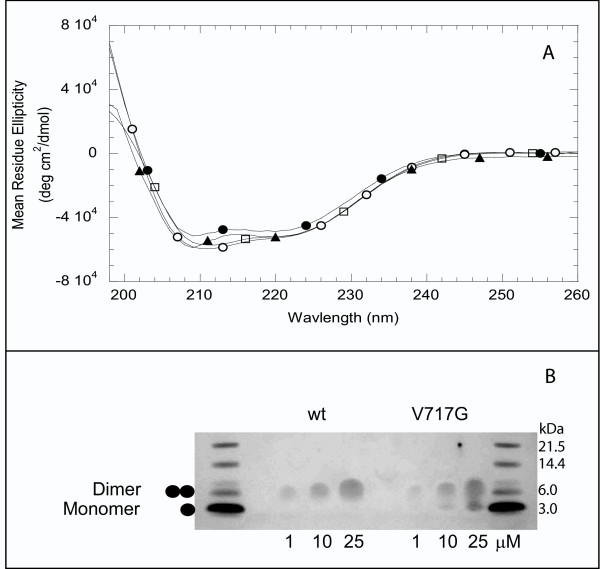
**APP-TM peptides adopt α-helical dimeric structure in SDS micelles**. **A**. Circular dichroism (CD) spectroscopy of APP-TM peptides in the presence of 20 mM SDS at pH 7. The CD spectra of wild-type (●), V717G (○), V717F (□) and T714I (▲) show that all APP-TM peptides form similar highly helical structures in 20 mM SDS. **B**. Oligomeric state of APP-TM peptides at different concentrations as determined by SDS-PAGE. Wild-type APP-TM peptides were predominantly dimeric under all concentrations tested, while V717G APP-TM peptides formed monomer-dimer mixtures across the same concentration range.

We examined the secondary structure and oligomerization states of wild-type APP-TM peptides and the following FAD mutant derivatives: T714I, V717G, and V717F APP-TM peptides. All APP-TM peptides were capable of inserting into SDS micelles and adopted similar highly α-helical structures (Fig. 1A). SDS-PAGE analysis showed that wild-type APP-TM peptides formed stable dimers, while the V717G peptides populated both monomer and dimer states under the same conditions (Fig. 1B). T714I and V717F APP-TM peptides were predominantly monomeric under these conditions (data not shown).

**Figure 2:**
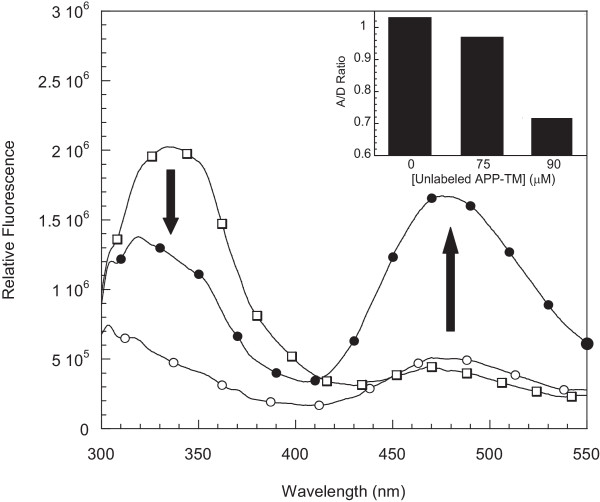
**APP-TM peptides oligomerize within phospolipid bilayers**. Different forms of fluorescent donor (Trp) and acceptor (Edans) labeled APP-TM peptides were incorporated in DOPG phospholipid vesicles. Purified peptide-incorporated vesicle fractions were subjected to fluorescence measurements. Typical donor quenching and acceptor sensitization was observed and is indicative of FRET. The fluorescence spectra of donor-labeled APP-TM wild-type peptide (□), acceptor-labeled APP-TM wild-type peptide (○), and a mixed sample (●) are shown. Note: arrows show donor quenching and acceptor sensitization and indicate that the peptides oligomerize in phospholipid bilayers. **Inset: FRET decreases with increasing concentration of unlabeled peptide in phospholipids bilayers**. Equimolar mixtures of donor- and acceptor-labeled APP-TM peptides were mixed with increasing concentrations of unlabeled peptide. Total peptide concentration was held constant at 100 μM. The unlabeled peptides act as competitors and reduce the likelihood of forming dimers containing both donor- and acceptor-labeled peptides, which results in a decrease in FRET and A/D ratio.

To determine whether the APP-TM peptides show similar behavior in phospholipid bilayers as they do in SDS micelles, we examined APP-TM peptides incorporated into dioleoyl phosphatidylglycerol (DOPG) vesicles using fluorescence resonance energy transfer (FRET) analysis. The compositions of these samples were as follows: (a) vesicles containing donor-labeled and unlabeled peptide mixtures, (b) vesicles containing acceptor-labeled and unlabeled peptide mixtures, and (c) vesicles containing equimolar mixtures of donor- and acceptor-labeled peptides. The fluorescence emission spectra of these peptide preparations are shown in Figure 2. In the preparation containing both donor and acceptor, the emission of the Trp fluorophore is significantly quenched compared to the emission of vesicles with only Trp-labeled peptide. The EDANS emission, on the other hand, is significantly enhanced in the mixed fluorophore preparation relative to the emission with acceptor only. Certain unavoidable factors, such as differential incorporation of peptides in vesicles, trapping of peptides within vesicle lumens, and asymmetric distribution of parallel and antiparallel orientations of peptides in the bilayer, can affect the fluorescence emission of the donor and acceptor. However, the observed donor quenching and acceptor sensitization are hallmark signatures of FRET and indicate that the peptides incorporated in DOPG vesicles are oligomeric in structure. A quantitative measure of the extent of FRET was obtained by measuring the ratio of acceptor fluorescence to donor fluorescence (i.e. A/D ratio; see Materials and Methods). To confirm that the observed spectral changes are caused by FRET, the A/D ratio was measured in the presence of increasing amounts of unlabeled peptide. The addition of excess unlabeled peptide caused a continuous decrease in the A/D ratio (Fig. 2 inset). This reduction in FRET suggests that the unlabeled peptides were forming mixed oligomers with the labeled peptides and preventing association between donor and acceptor fluorophores. The similarity of the FRET results obtained with DOPG vesicles and those obtained with SDS micelles (see below) suggests that the APP-TM peptides associate into similar structures in both systems.

### Model of the predicted structure of the APP-TM dimer

**Figure 3:**
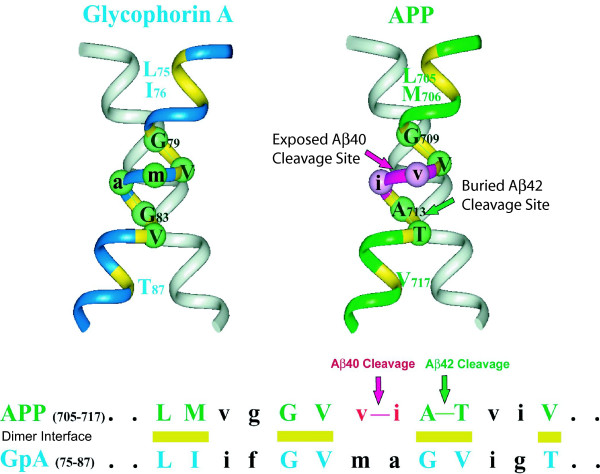
**Model of Alzheimer precursor protein transmembrane (APP-TM) dimer**. The solid state NMR structure of the glycophorin A (GpA-TM) dimer, residues 75–87 (left), and the predicted APP-TM dimer, residue 705–717 (right) are compared. GpA-TM dimer contains the central G79VXXGV84 residues as the packing contacts, whereas APP-TM dimer packing utilizes G709VXXAT714 residues. Sequence alignment of APP-TM and GpA-TM (bottom) shows that residues comprising the packing interface are comparable in both sequences and denoted by capital letters. Exposed residues are shown as lowercase letters.

We predicted a model for the APP-TM dimer structure using a previously described method that has proven to be remarkably accurate for modeling homo-oligomeric TM domains [6]. The predicted structure displays many similarities with the NMR structure of glycophorin A (GpA) [7] (Fig. 3). The GpA-TM dimer packing interface utilizes the sequence G_79_VXXGV_84_, while APP-TM dimer packing utilizes G_709_VXXAT_714_. Sequence alignment of APP-TM and GpA-TM (Fig. 3) shows that residues at the dimer interface are comparable in both sequences. A notable feature of the APP-TM model is that majority of the FAD mutations in the APP-TM domain are located within the predicted dimer interface (Table 1).

**Table 1:** Location of FAD mutations within the APP-TM sequence

APP (709–717)	**709**	**710**	711	712	**713**	**714**	715	716	**717**
Wild-type sequence	**G**	**V**	v	i	**A**	**T**	v	i	**V**
FAD mutations in the APP-TM domain					**T**	**I**	m	v	**F**
						**A**	a	t	**G**
									**I**
									**L**
									

The segment of the APP-TM domain comprising residues 709 to 717 is shown. Residue positions that are capitalized and bolded represent positions predicted to form the dimer interface. Note that the majority of FAD mutations in the APP-TM domain occur in dimer interface positions. APP mutation references [8, 16-22].

### Dimer stability of wild-type and FAD mutant derivatives of APP-TM peptides

FRET analysis of wild-type APP-TM peptide and FAD mutant derivatives also produces the signature hallmarks of FRET (i.e. donor quenching and acceptor sensitization). This indicates that wild-type and mutant peptides oligomerize in both SDS detergent micelles (data not shown) and phosphatidylglycerol bilayers (Fig. 2). SDS-PAGE showed that V717G populated both monomer and dimer states under conditions where wild-type formed stable dimers (Fig. 1B), suggesting that the FAD mutations reduce the stability of APP-TM dimers.

**Figure 4:**
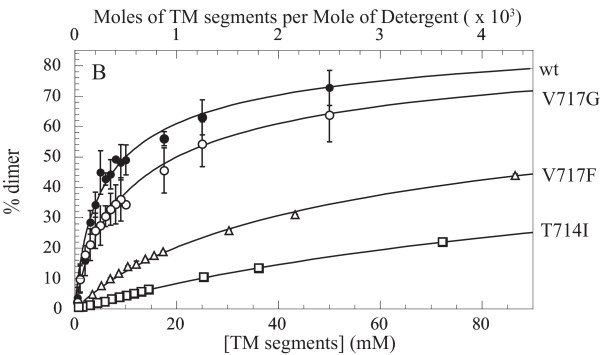
**Apparent dissociation constants of APP-TM peptides in SDS micelles**. The apparent dimer dissociation constants for wild-type and mutant APP-TM peptides were determined by examining the concentration dependence of FRET in equimolar mixtures of donor- and acceptor-labeled peptides. The FRET signals were converted to percent dimer and plotted against total APP-TM peptide concentration. The apparent dissociation constants are as follows: 10.3 μM (wild-type), 20.4 μM (V717G), 125 μM (V717F) and 395 μM (T714I).

To quantify whether other FAD mutations reduce the stability of APP-TM dimers, the apparent dimer dissociation constants of all of the peptides were obtained through analysis of the concentration-dependence of FRET (Fig. 4). All mutations tested caused an increase in the apparent dissociation constants from 10.3 μM (wild-type) to 20.4 μM (V717G), 125 μM (V717F) and 395 μM (T714I) (average error 3.6%). Interestingly, T714I, which causes the largest increase in Aβ42/40 ratio in FAD [8] is the least stable dimer.

### Mixed oligomerization of wild-type and FAD mutant APP-TM peptides

**Figure 5:**
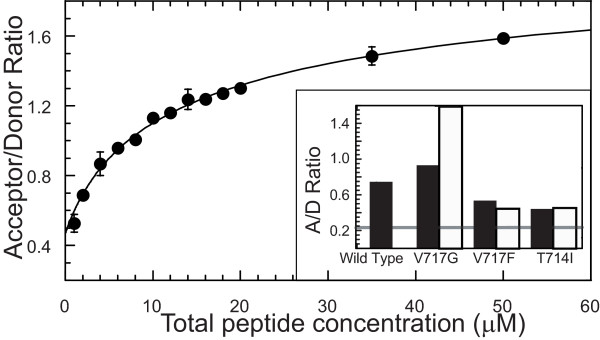
**Hetero-oligomerization of wild-type and mutant APP-TM peptides monitored by FRET**. Trp-labeled wild-type APP-TM peptides were incubated with equimolar amounts of Edans-labeled V717G APP-TM peptides in the presence of 20 mM SDS. The A/D ratio is plotted as a function of total peptide concentration. Inset: The solid bars represent the A/D ratio of APP-TM homodimers at 50 μM total peptide concentration. The open bars represent the A/D ratio of wild-type and mutant heterodimers at 50 μM total peptide concentration. The gray line indicates the A/D ratio for a mixture of monomeric peptides.

With the autosomal dominant inheritance pattern of APP-FAD [9] both wild-type and mutant APP are expressed in heterozygotes. Combined with the dimerization propensity of APP, there is a possibility that heterodimerization of mutant and wild-type APP could contribute to FAD. To determine whether the APP-TM domain may mediate the formation of hetero-oligomers, we employed FRET experiments using mixed preparations of wild-type and mutant APP-TM peptides. The FRET signal (i.e. the A/D ratio) between the Trp fluorophore on wild-type APP-TM and the EDANS fluorophore on V717G APP-TM peptides was measured in a series of samples. These samples contained increasing amounts of wild-type and mutant peptide but a constant 1:1 ratio of wild-type to mutant peptide was maintained (Fig. 5). The results show that the A/D ratio increases with total peptide concentration and shows signs of saturation above 50 μM total peptide concentration. While it is not possible to extract the apparent equilibrium constant for hetero-oligomerization from this data, the similarities in the shapes of the FRET curves for self-association (Fig. 4) and mixed-association (Fig. 5) suggest that both processes are energetically similar. To determine whether the wild type peptide can form mixed oligomers with the other FAD mutant peptides, the A/D ratio of APP-TM homodimers were compared with the A/D ratio of equimolar mixtures of wild-type and mutant peptides at 50 μM total peptide concentration (Fig. 5 inset). The A/D ratios of the mixed peptide samples are significantly higher than 0.24, which is the baseline A/D ratio for non-interacting peptides, indicating that wild-type APP-TM can hetero-oligomerize with all mutant APP-TM peptides tested. The A/D ratios of the homodimeric samples differ from those of the mixed peptide samples. These differences may be the result of altered orientation of the fluorophores in the mixed samples leading to changes in the dipole moment orientation factor and FRET efficiency.

### Comparison of dissociation constants to clinical pathology of AD patients

**Figure 6:**
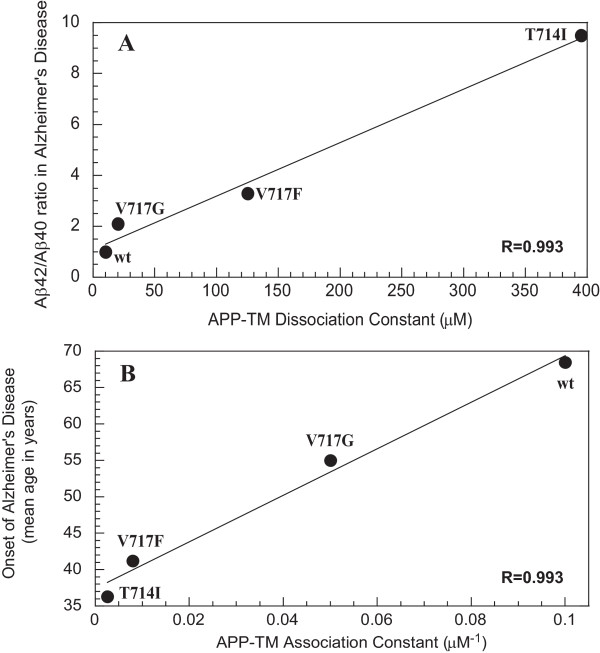
**Comparison to the clinical pathology of AD patients**. The dimerization constants of the APP-TM peptides were compared to the **A) **Aβ42/Aβ40 ratio and **B) **mean age of onset. The mean age of onset was obtained from all available families [2]. The dissociation constants are positively correlated to the Aβ42/Aβ40 ratios and similarly association constants are equally correlated to the mean age of onset.

The dissociation constants of the APP-TM variants were compared to the Aβ42/Aβ40 ratio and the mean age of onset for the different mutations (Fig. 6). Mean age of onset for the different mutations was calculated using all available families [2]. Aβ42/Aβ40 ratios were obtained from published studies using familial Alzheimer's disease brain or cell cultures [10-12]. The dissociation constants exhibited a direct correlation to the critical Aβ42/Aβ40 ratio (R = 0.993); the mutation-induced increases in the dissociation constants were mirrored by increases in the Aβ42/Aβ40 ratio in these FAD cases. Strikingly, the APP-TM association constant were also directly correlated to the mean age of onset of AD (R = 0.993), that is, the weaker the association constant, the earlier the age of onset of AD symptoms.

### Inhibition of Aβ production by APP-TM peptides in HEK293 cells overexpressing APP and γ-secretase components

We examined whether the addition of APP-TM peptides to cells expressing APP interferes with the production of Aβ40 and Aβ42. As it has been shown that cationic peptides, such as those derived from the HIV-TAT protein, can be taken up spontaneously by cells [13,14], we reasoned that the cationic APP-TM peptides may also be taken up by similar mechanisms. Once inside the cell, these peptides, which have identical TM sequences to endogenous APP, could potentially interfere with the γ-secretase activity via a competitive inhibitor mechanism and alter production of Aβ42 and Aβ40. HEK293 cells overexpressing APP and γ-secretase components were incubated with APP-TM peptides and the conditioned media was assayed for Aβ42 and Aβ40 levels using a commercial assay (BioSource). The APP-TM peptides, which share sequence identity with Aβ42 and Aβ40, do not interfere in this assay (see Materials and Methods). Only wild-type and V717G peptides could be examined in this experiment because the other mutant peptides, V717F and V714I, were only soluble in the presence of SDS, which is toxic to cells.

**Figure 7:**
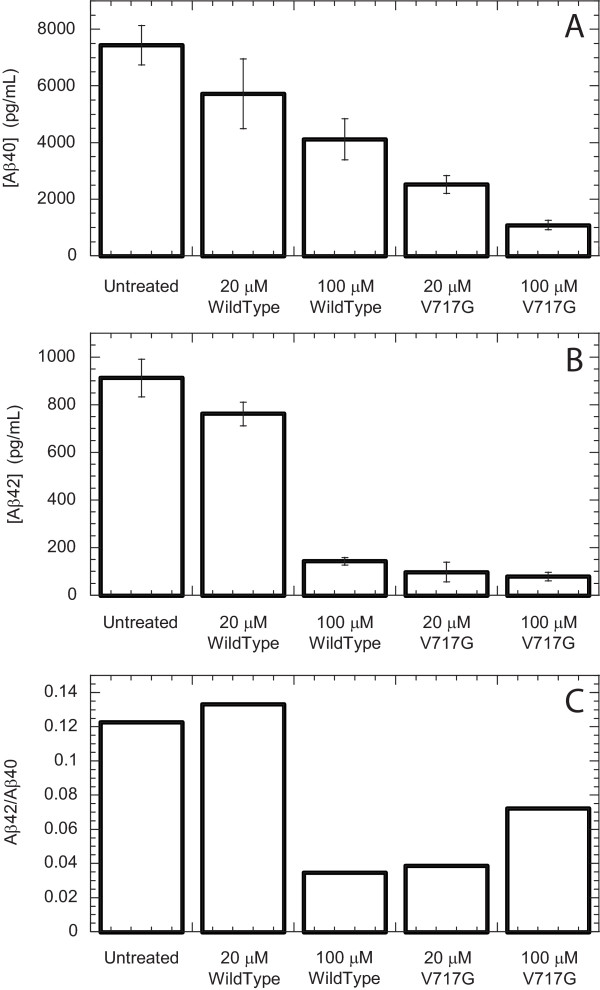
**Inhibition of Aβ production by APP-TM peptides in cells stably overexpressing APP**. HEK293 cells stably expressing Swedish mutant APP and the γ-secretase components were incubated overnight with APP-TM peptides. An Aβ ELISA immunoassay was used to quantify the concentration of Aβ40 (**A**) and Aβ42 (**B**) produced and secreted into the conditioned cell culture media. The effect of the APP-TM peptides on Aβ42/40 ratio is shown in **C**.

**Figure 8:**
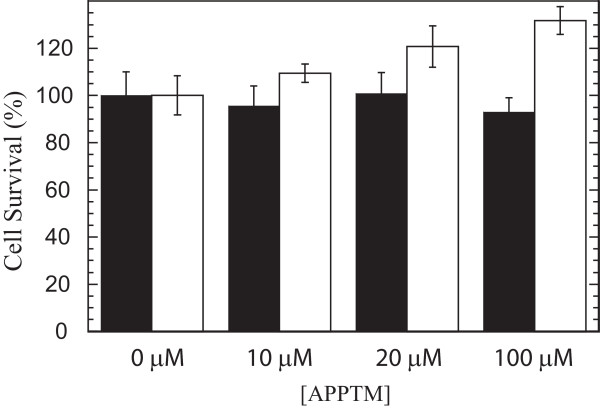
**APP-TM peptides are not toxic to HEK293 cells stably overexpressing APP and γ-secretase components**. HEK293 cells stably expressing Swedish mutant APP and the γ-secretase components were incubated overnight with APP-TM peptides. The SRB cytotoxicity assay was used to determine whether wild-type (black bars) and V717G (clear bars) affected cell survival. Note that neither peptide caused a reduction in cell survival.

**Figure 9:**
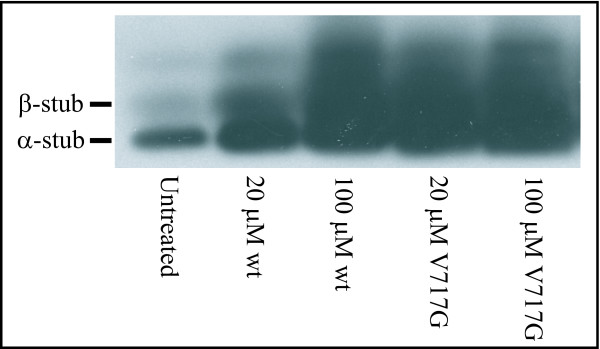
**Addition of APP-TM peptides to HEK293 cells affects APP processing and leads to an increase in γ-secretase substrates**. HEK293 cells were incubated overnight with APP-TM peptides. The cells extracts were separated by SDS-PAGE and probed by western blotting with an APP C-terminal antibody (Sigma). See Blue plus-2 (Invitrogen) molecular weight markers were used. Total protein concentration was the same in each lane. There is a noticeable increase in the amount of γ-secretase substrates (α- and β-stubs) relative to untreated cells.

The addition of wild-type and V717G peptides to the HEK293 cells resulted in a drastic reduction in the levels of Aβ relative to control samples and displayed a strong concentration dependence (Fig. 7A,B). The ability of the V717G peptide to suppress Aβ production was greater than that of the wild-type peptide. Both peptides had a greater inhibitory effect on Aβ42 production (Fig. 7B) than on Aβ40 production (Fig. 7A). Because of their greater inhibitory effect on Aβ42 production, both peptides caused a reduction in the Aβ42/40 ratio relative to control samples (Fig. 7C), which were comparable to *in vivo *Aβ42/Aβ40 ratios [15]. Importantly, the reduction in Aβ production is not caused by any cytotoxic activity of the peptides. Examination of the effect of APP-TM peptides on the viability of these HEK293 cells demonstrated that they do not reduce cell viability (Fig. 8). Analysis of the cell extracts revealed an accumulation of the α- and β-stub fragments of APP, which are γ-secretase substrates. Accumulation of γ-secretase substrates is evidence of γ-secretase inhibition (Fig. 9). While the mechanism by which these peptides reduce Aβ production requires further investigation, their action may be caused by interactions between the γ-secretase complex and monomeric/dimeric forms of the APP-TM peptide or heterodimeric complexes of the peptides with endogenous APP (see below).

## Discussion

Our studies indicate that APP dimerization propensity, measured *in vitro*, is correlated with two previously identified *in vivo *pathological features of FAD: the Aβ42/40 ratio and the age of onset of AD symptoms. While our biophysical studies indicate that APP-TM peptides form dimers that are sensitive to APP-FAD mutations, validation of the predicted structure will require high-resolution techniques, such as NMR spectroscopy. However, when the locations of FAD mutations in the TM domain of APP are mapped onto our predicted structure of the APP-TM an intriguing feature arises. Out of the 11 known FAD mutations in the APP-TM domain, 7 occur within the predicted dimer interface and the remaining 4 are adjacent to the interface (Table 1) [8,16-22]. These mutations are ideally located to disrupt APP dimerization. Our experiments on FAD mutant derivatives indicate that such mutations can in fact result in loss of APP-TM dimerization (Fig. 4). The dissociation constants of our APP-TM peptides may differ from full-length APP; however, what is important is the relative difference between the wild type dissociation constant and that of the FAD APP-TM variant. Previoulsy, Aβ42/Aβ40 ratios determined using primary culture neurons were compared to the mean age of onset and found to exhibit a strong inverse correlation [10]. Here we compared our dissociation constants to these two important clinical features of AD. Interestingly, our *in vitro *K_d _measurements are directly correlated to the Aβ42/Aβ40 ratios and the *in vitro *K_a _measurements are equally correlated to the mean age of disease onset. These correlations suggest that the relative production of Aβ42 increases as the equilibrium between dimeric and monomeric APP shifts toward monomer. The exact mechanism responsible for causing the shift in Aβ42/Aβ40 ratios between wild-type and FAD mutants is currently unclear. It is likely, however, that the preferential cleavage at either the Aβ40 or Aβ42 sites is related to the process by which the γ-secretase complex recognizes monomeric versus dimeric APP.

There is evidence that APP dimers are physiologically relevant as it has previously been shown that APP dimers exist *in vivo *and that these dimers are substrates for β- and γ-secretase. Scheuermann et al [4] have provided direct biochemical evidence for the presence of APP dimers in cellular environments using chemical cross-linking experiments on full-length and deletion mutants of APP. They identified two extracellular domains in APP that mediate dimerization. A third dimerization domain was detected and its location was determined to be between the start of the TM domain and the end of APP at the C-terminus. It is likely that the APP-TM domain studied here represents the precise location of this third dimerization domain. The X-ray crystal structure of the extracellular E2 domain of APP [23] demonstrates that it is dimeric and likely corresponds to the first extracellular dimerization domain detected by Scheuermann et al [4]. Scheuermann *et al*. also developed a site-directed obligate dimer of APP where engineered Cys residues placed at the N-terminal of the TM domain tether together two monomers with a disulfide bond. When expressed in cells, this obligate APP dimer is efficiently cleaved by β- and γ-secretases to form Aβ. There is, therefore, significant biochemical data that indicates that APP dimers exist *in vivo *and are substrates for β- and γ-secretases.

Dimerization appears to be a common theme among several membrane proteins involved in Aβ metabolism and AD. Both BACE [24], the membrane-bound protease responsible for β-site cleavage of APP, and presenilin 1 [25], the catalytic component of γ-secretase, are dimeric in their native membrane environments. While the dimeric nature of these proteases does not provide evidence for APP dimerization, this common theme is nevertheless noteworthy.

Recently, Marchesi proposed a novel mechanism of Aβ toxicity that involves homo- and hetero-dimerization of α-helical membrane segments [26]. Marchesi proposes that a certain fraction of Aβ molecules generated by β- and γ-secretases remain inserted in the membrane bilayer in a helical conformation and can utilize its GXXXG/A motif to form homodimers or heterodimers with other GXXXG/A membrane proteins, such as APP or Aph1. It is speculated that accumulation of homo- and hetero-dimers of Aβ interferes with biochemical processes that occur in the membrane including receptor, channel, and secretase activities. This interference may lead to downstream events that culminate in eventual neuronal dysfunction. While Marchesi's proposal and the hypothesis reported here are fundamentally different, they both invoke the contribution of homo- and hetero-typic interactions of membrane proteins in AD pathology. The heterotypic association of wild-type and mutant APP-TM peptides that we have observed (Fig. 5) reinforces the idea that such interactions could occur with other molecules, such as Aβ and Aph1.

The addition of APP-TM peptides to Aβ-producing HEK293 cells causes dramatic reductions in Aβ production, and intriguingly, the V717G mutant caused significantly greater inhibition of Aβ production than the wild-type peptide (Fig. 7). While elucidation of the mechanism of inhibition requires further study, several feasible possibilities arise involving the interaction of the peptides with the γ-secretase complex. The γ-secretase complex can potentially interact with monomeric or dimeric forms of the APP-TM peptides or form heteromeric complexes of peptide with endogenous APP. Both monomeric and homodimeric forms of the APP-TM peptides could act as competitive inhibitors of γ-secretase leading to the observed accumulation of γ-secretase substrates (i.e. α- and β-stubs) and decrease in γ-secretase products (i.e. Aβ). Mutational and chemical crosslinking studies are underway to test these hypotheses. It should be noted that peptides analogous of the APP-TM domain that are too short to span the plasma membrane have also been shown to inhibit γ-secretase activity [27].

## Conclusion

We have provided in evidence using a cell culture model that the extent of APP dimerization can determine the Aβ42/40 ratio and disruptions of dimerization induced by at least one FAD mutation in the APP-TM domain increases the Aβ42/40 ratio. These studies have to be replicated *in vivo *to substantiate the hypothesis that dimeric APP causes preferential production of the less neurotoxic Aβ40. A detailed understanding of the pathological and physiological processing of APP would be a valuable asset in the development of novel therapeutic strategies, such as small molecule stabilizers of the dimeric form of APP, aimed at controlling Aβ production.

## Methods

### APP-TM peptide sequences

Wild-type: Lys-tag(W/E_EDANS_)GAIIGLMVGGVVIATVIVITLVML Lys-tag

V717G: Lys-tag(W/E_EDANS_)GAIIGLMVGGVVIATVIGITLVML Lys-tag

V717F: Lys-tag(W/E_EDANS_)GAIIGLMVGGVVIATVIFITLVML Lys-tag

T714I: Lys-tag(W/E_EDANS_)GAIIGLMVGGVVIAIVIGITLVML Lys-tag

The sequences of the APP-TM peptides are shown above, and the central block of residues given in the one-letter code correspond to residues 700 to 723 of human APP and contain the TM segment. All peptides contain N- and C-terminal oligo-lysine tags, which have been shown previously to increase aqueous solubility of TM segments [28]. Two versions of each peptide were synthesized. The first version contained a Trp residue that followed the N-terminal Lys-tag; the second version differed from the first in that a Glu residue with the fluorescent group EDANS covalently linked to its sidechain (Glu-EDANS; Molecular Probes) was used instead of the Trp residue. The fluorescence of the Trp residue and the EDANS group were used to follow dimerization of the peptides through fluorescence experiments discussed below.

All peptides were prepared by solid-phase synthesis on a PerSeptive Biosystems 9050 Plus peptide synthesizer as previously described [29]. The peptides were cleaved with 88% trifluoroacetic acid/5% phenol/5% distilled H_2_O/2% tri-isopropylsilane (v/v). Cleaved peptides were precipitated with ice-cold diethyl ether, dissolved in distilled H_2_O and lyophilized. Crude peptide powder was dissolved in water and purified by reverse phase C4-HPLC. Mass spectroscopy was used to confirm the molecular weight of the purified peptide. The stock peptides solutions were stored in distilled H_2_O at 4°C.

### Structural modeling of APP-TM oligomerization

Detailed modeling procedures for TM helix oligomerization are described elsewhere [6,30]. The APP-TM sequence was built into uniform α-helices with backbone torsion angles of Φ = -65° and ψ = -40°. Sidechain rotamers were chosen using the backbone-dependent rotamer library program SCWRL [31]. The Monte Carlo (MC) search procedures and parameters for potential helix packing were described previously [6]. Homo-dimeric APP-TM models were selected from four hundred independent MC simulations. The selected model structures were clustered by Cα RMSD using the NMRCLUSTER program [32]. The median model from the largest cluster was selected as our final predicted structure.

### Fluorescence resonance energy transfer of APP-TM peptides in the prescence of phospholipids bilayers and SDS micelles

DOPG vesicles with incorporated peptides were prepared via sonication of dried lipid films in the presence of peptide solution at a molar ratio of 1 mole of peptide: 20 moles of DOPG as previously described [33]. The samples contained 50 mM Tris, 10 mM NaCl (pH 7.0) as the buffer. Gel filtration experiments on these samples indicated that the majority of the peptides were incorporated into the vesicles and absorbance measurements indicated that similar amounts of donor- and acceptor-labeled peptides were incorporated. Purified peptide-incorporated vesicle fractions were used for fluorescence measurements.

Mixed peptide-SDS micelles were prepared by the addition of concentrated SDS to peptide solutions, such that the final concentrations were 20 mM SDS, 40 mM Tris, 8 mM Acetate (pH 7.0) and 0 – 90 μM peptide. The samples were sonicated and incubated overnight in the dark at room temperature before making fluorescence measurements.

Fluorescence measurements were made at room temperature using a PTI QM-1 fluorescence spectrophotometer. A quartz cuvette with a 2 mm excitation pathlength and a 1 cm emission pathlength was used. The excitation wavelength was 285 nm and the bandpass was 4 nm. We obtained a quantitative measure of the degree of peptide oligomerization by integrating the EDANS emission signal from 472–496 nm and dividing this value with the integrated Trp emission signal from 350–375 nm. This ratio of acceptor fluorescence to donor fluorescence is denoted either as Acceptor/Donor ratio or A/D ratio and can be used to compare peptide oligomerization at varying concentrations.

### SDS-PAGE

Samples contained 20 mM SDS, 40 mM Tris, 8 mM Acetate (pH 7.0), 5% glycerol, and 0 – 25 μM peptide. They were boiled for 5 minutes and subjected to SDS-PAGE using precast 4–12% Nupage gels (Invitrogen) and a standard running buffer. SeeBlue^®^Plus2 molecular weight markers from Invitrogen were used as standards.

### Circular dichroism (CD) spectroscopy

Circular Dichroism (CD) spectra were recorded on an Aviv-62DS CD-spectrometer at 25°C. Spectra were obtained from 260 nm to 198 nm with a 1.0 nm step size, a 1 nm bandwidth, a 1 mm pathlength and an averaging time of 8 seconds. The peptide concentration was 80 μM.

### Data and statistical analysis

The apparent dissociation constants were determined using an equation derived from first principles of monomer-dimer equilibrium, where K_d _= [A]^2^/[A_2_]. The fluorescence acceptor-donor ratio was plotted against total peptide concentration and fit to the following equation:

$$[A_{T}]=K_{d}\frac{\left(\frac{F_{obs}-F_{\min}}{F_{\max}-F_{\min}}\right)}{2{\left(1-\frac{F_{obs}-F_{\min}}{F_{\max}-F_{\min}}\right)}^{2}}$$

Where A_T _= total APP-TM peptide concentration, F = A/D ratio, fraction dimer = (F_obs_-F_min_)/F_max_-F_min_), and % dimer = 100% (fraction dimer). The goodness-of-fit to the model were assessed using the chi-squared test, nonlinear least squares correlation, and examination of residuals.

### HEK293 cell culture, cytotoxicity and Aβ ELISA

Stably transfected HEK293 cells overexpressing the Swedish double mutant (K670N/M671L) of APP751, presenilin 1, nicastrin, PEN-2 and APH-1 were grown to confluency in DMEM media containing 10% fetal bovine serum [34]. The cells were grown for another 48 hours with the media changed twice at equivalent time intervals. Cells were then incubated overnight in fresh media containing 0, 20 or 100 μM APP-TM peptides. The media was collected and subjected to an immunoassay to determine the Aβ42 and Aβ40 concentrations. The ELISA BioSource immunoassay *human ***β-amyloid 1–40/1–42 **colorimetric kit (catalogue number KHB3482/KHB3481) was used and the standard protocol was followed. This sandwich ELISA technique makes use of an immobilized Aβ antibody that binds both Aβ40 and Aβ42 at an N-terminal epitope. The bound Aβ40 and Aβ42 are then detected using a C-terminal antibody specific for either Aβ40 or Aβ42. The APP-TM peptides, which share sequence identity with both Aβ40 and Aβ42, do not interfere in this analysis because they lack the N-terminal epitope responsible for capturing Aβ40 and Aβ42 from the media. Therefore, this assay is ideally suited to detect any disturbances in the normal processing of endogeneuous full length APP in the presence of APP-TM peptides.

Sulforhodamine B (SRB) cytotoxicity assay was used to assess whether the APP-TM peptides possessed any intrinsic toxicity. Stably transfected HEK293 cells overexpressing Swedish-mutant APP751 and γ-secretase components were plated at 15,000 cells/well in DMEM media containing 10% fetal bovine serum. The cells were grown for another 48 hours with the media changed twice at equivalent time intervals. Cells were then incubated overnight in fresh media containing 0, 10, 20, and 100 μM APP-TM peptides, and the SRB cytotoxicity assay was performed as previously described [35].

## Authors' contributions

PMG, MG, and RM carried out the molecular biophysical and cell culture studies. SK carried out the computational studies. PEF and JM oversaw the cell culture and immuno-assay experiments. PMG, SK, MG, RM, JB, and AC conceived of the study, and participated in its design and coordination. All authors read and approved the final manuscript.
